# A case of left foot drop as initial symptom of granulomatosis with polyangiitis: Triggered by COVID‐19 disease?

**DOI:** 10.1002/ccr3.6418

**Published:** 2022-10-13

**Authors:** Marjolaine Weynand, Ioannis Raftakis, Yassine Mohammad Chérif, Sophie Lecomte, Valérie Badot

**Affiliations:** ^1^ Department of Rheumatology Brugmann University Hospital, Université Libre de Bruxelles (ULB) Brussels Belgium; ^2^ Department of Anatomical Pathology Brugmann University Hospital, Université Libre de Bruxelles (ULB) Brussels Belgium

**Keywords:** ear, nose and throat, neurology, ophthalmology, rheumatology

## Abstract

In Granulomatosis with polyangiitis (GPA), involvement of the peripheral nervous system is frequent but its occurrence as an initial presentation is unusual. This case highlights the importance of this occurrence to permit an early diagnosis. Moreover, GPA started after a coronavirus disease 2019 infection and could have been induced by this.

## Abstract 2

The physicians should recognize peripheral neuropathy as one of the first presentations of GPA, even if it’s rare, in order to permit an early diagnosis. Moreover, this case complements previously reported cases of COVID‐19‐induced auto‐immune disease.

## 1 INTRODUCTION

In recent years, various post coronavirus disease 2019 (COVID‐19) syndromes have been described. Moreover, many viruses have been implicated in the pathogenesis of auto‐immune diseases, including vasculitis. Here, we report a case of granulomatosis with polyangiitis onset following a COVID‐19 disease with an initial presentation of left drop foot.

Granulomatosis with polyangiitis (GPA) is an ANCA‐associated vasculitis (AAV) belonging to the primary systemic vasculitis and systemic necrotizing vasculitides (SNV). The disease is characterized by necrosis of small blood vessels (capillaries, venules, arterioles, and small arteries) and by granulomatous formation in multiple organs.^1^ It is a rare disease with an annual incidence of about 10 cases per million inhabitants in northern Europe.^1^ If untreated, the mortality rate at 1 year is about 70%.^2^ This highlights the importance of a prompt diagnosis, and needs to raise awareness of the most uncommon presentations. It usually presents with ear–nose–throat, pulmonary or/and renal involvement.^1^ Nasal and paranasal sinus disorders are observed in 90% of the cases, and are the most common primary manifestations.^1^ Pulmonary infiltrates or nodules come second among the most frequent manifestations.^1^ Although nervous system involvement is rare at the initial stage,^3^ neuropathy is reported during the course of the disease in 17% of cases.^4^ The typical presentation is a mononeuritis multiplex, leading to motor and sensory deficits.^3^, ^5^ On the contrary, an isolated peripheral neuropathy, like the one described here, is an unusual finding in GPA.^3^

## 2 CASE PRESENTATION

**FIGURE 1:**
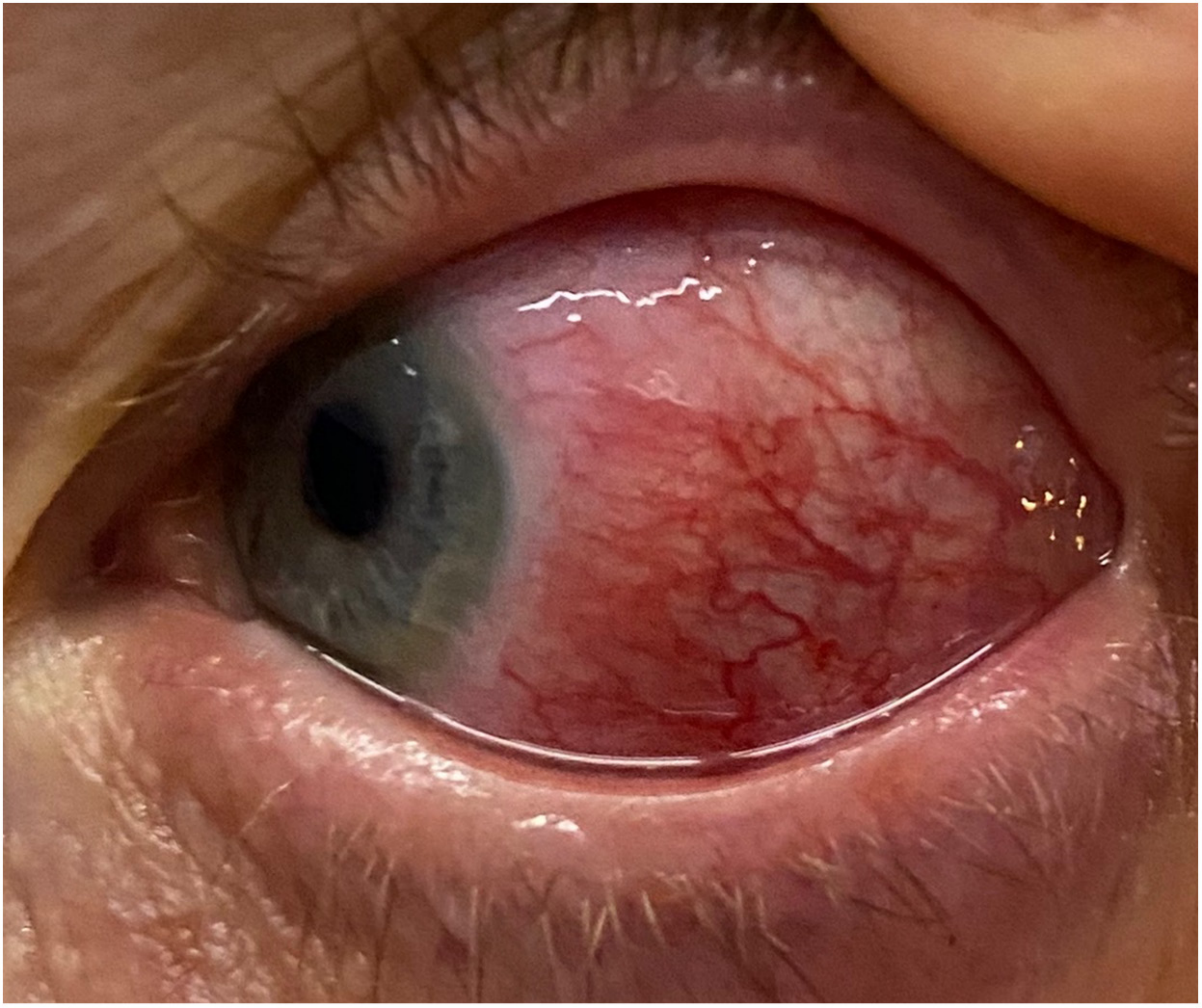
External appearance of the diffuse anterior scleritis with widespread inflammation of sclera. The scleral plexus remains visible after phenylephrine.

In January 2021, a 51‐year‐old woman was admitted in our department with a worsening chronic polyarticular pain and fatigue but also with a left drop foot that had begun few weeks earlier associated with burning paresthesia. Prior to her admission, she reported redness of the left eye with ocular pain and photophobia (Figure 1). She had no fever, no photosensitivity rashes, no Raynaud phenomenon and no swelling joints. Her past medical history included diabetes, hypertension, osteoarthritis, and chronic non‐allergic rhinitis. We noted a diagnosis of mild‐form COVID‐19 infection 3 months prior without acute respiratory distress syndrome or multiorgan failure. Her chronic treatment included Bisoprolol and Metformine. She had no smoking history, and did not take any drugs.

Physical examination found the left drop foot, and the conjunctival redness of the left eye. Neurological examination revealed a weakness in the dorsiflexion of the left foot, and hypoesthesia of the lateral surface of the distal leg. The rest of her examination, including deep tendon reflexes, was normal. The ophthalmological examination with slit‐lamp revealed a diffuse anterior scleritis of the left eye.

**FIGURE 2:**
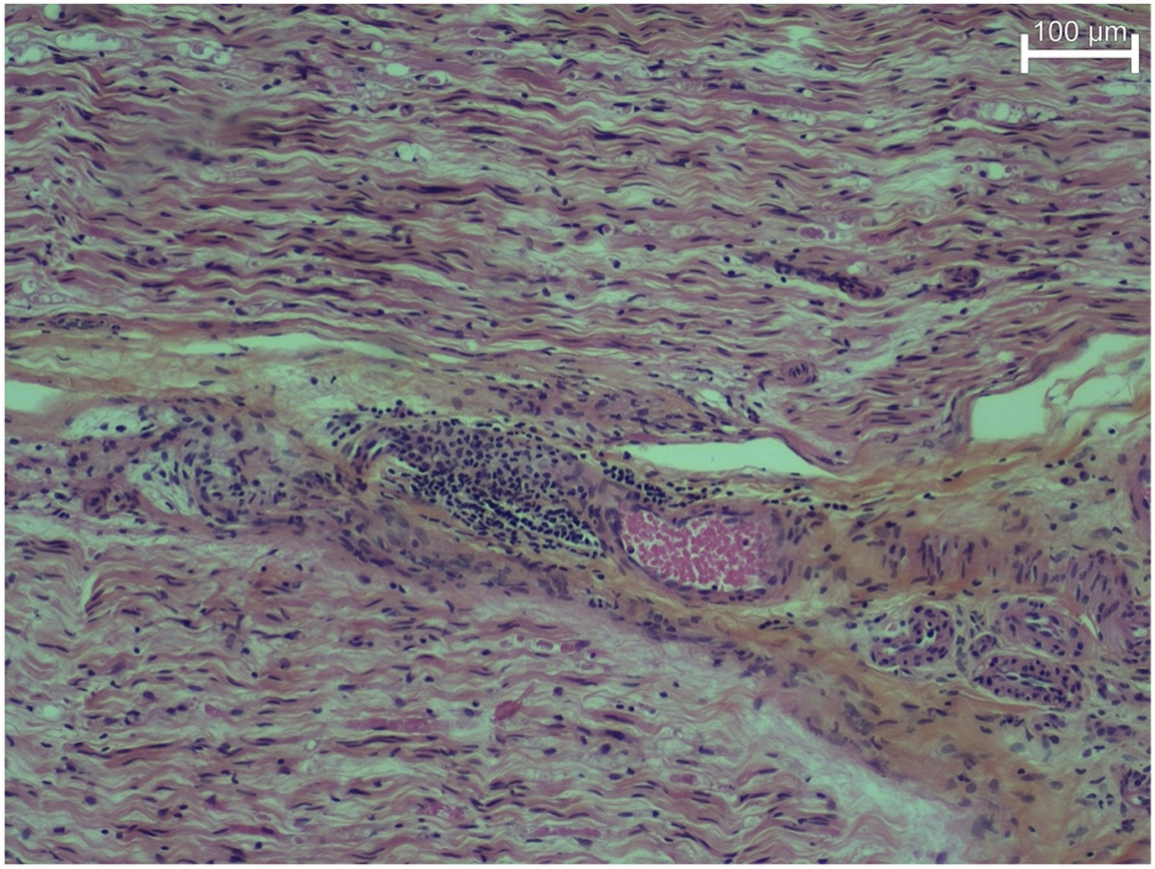
Superficial peroneal nerve biopsy with two nerve fascicles demonstrating perivasculitis with mononuclear cell infiltration surrounding small vessels.

An electromyography (EMG) and a lumbar magnetic resonance imaging (MRI) had been performed before the admission and were normal excepted a discal protrusion without radicular conflict. In the past, the patient was known for chronic moderate inflammatory blood test of unknown origin. Laboratory investigation confirmed again the elevation of the erythrocyte sedimentation rate (30/mm) and of C‐reactive protein (22 mg/L). The hemogram, renal, and liver functions were normal. She was negative for HIV, hepatitis viruses, and Lyme disease. Severe acute respiratory syndrome coronavirus 2 (SARS‐CoV‐2) antibodies were positive. The patient was tested negative for tuberculosis (TB) by an interferon‐*γ* releasing assay. Immunological tests were negative for antinuclear antibodies, anti‐cyclic citrullinated peptide antibodies, rheumatoid factor and for cryoglobulinemia. The complement components 3 (C3) and 4 (C4) levels were normal. However, cytoplasmic anti‐neutrophil cytoplasmic antibodies (c‐ANCA) titers were elevated (1/160) with presence of Anti‐proteinase‐3 (PR3) antibodies (744 U/ml [N < 20]). Blood cultures were negative. Urine analysis revealed microscopic hematuria, no red cell casts and no proteinuria. Ultrasound of the urinary tract and cystoscopy were normal. Computed tomography (CT) of the chest showed no abnormalities. A sinus CT described a moderate diffuse pansinusitis with mucosal thickening. A second nerve conduction study was performed and showed an isolated left fibular sensory‐motor neuropathy. On the left leg MRI, we visualized a neuritis of the fibular nerve with a denervation edema of the tibialis anterior and the extensor digitorum longus muscles. In the context of hematuria and suspicion of systemic disease, a renal biopsy was also performed and was negative. Nasal mucosa ulcers observed by endoscopy were biopsied and revealed only perivasculitis without necrotizing granulomas. Finally, a combined superficial peroneal nerve/peroneus brevis biopsy according to the electromyography revealed only a perivasculitis (Figure 2).

She was treated with topical prednisolone in her left eye, and a reduced‐dose regimen of systemic steroids treatment was initiated with rituximab infusion. This treatment showed a favorable outcome for our patient with pain resolution, and improvement of the drop foot. Physiotherapy was also started. The patient follows a rituximab‐based regimen for remission maintenance.

## 3 DISCUSSION

We report a case of GPA following a SARS‐CoV‐2 infection with an isolated drop foot as its first manifestation. Peripheral neuropathies are usual complications of primary systemic vasculitis,^3^, ^5^ but the presentation as first symptom^4^, ^6^ as an isolated mononeuropathy is quite rare particularly for GPA.^3^ Involvement of other nerves can occur gradually over the course of 1 year or simultaneously. The typical presentation is an acute or subacute painful multifocal neuropathy with asymmetric distribution (15%–24%).^5^ Polyradiculopathy, plexopathy, distal symmetric neuropathy, or exclusive motor neuropathy are less frequent.^1^, ^3^, ^5^, ^7^, ^8^ The peroneal nerve is most often affected (56%–96%), followed by the tibial (11%–86%), ulnar (24%–75%), median (9%–58%), and radial (4%–50%) nerves.^5^ Electrophysiological studies reveal in most cases multifocal axonal lesions with an asymmetric pattern.^8^ In our patient, initial nerve studies and EMG were normal, which is due to the short delay (2–3 weeks) between nerve fibers degeneration and the appearance of fibrillations.^9^ The second nerve studies and EMG of the four limbs described an isolated fibular sensory‐motor neuropathy.

It is important to keep in mind that peripheral neuropathy in the presence of elevated markers of inflammation should raise suspicion of a systemic illness.

ANCA testing plays an essential role in the diagnostic process of AAV. GPA is associated with a cytoplasmic pattern of ANCA (cANCA) staining on immunofluorescence (IF) which have an affinity to PR3. Sensitivity of ANCA testing for patients with systemic GPA is around 78% to 96%.^10^ The combination of IF and enzyme‐linked immunosorbent assay (ELISA) increases this sensitivity.^10^ Among patients presenting with vasculitic neuropathy, sensitivity of ANCA testing is around 75%.^7^ In this case, nerve involvement was rapidly followed by an unilateral diffuse anterior scleritis, another unusual manifestation as an initial presentation. In GPA, ocular and orbital (like pseudotumor) symptoms are found in 15% of cases at first assessment, and in approximatively 23 to 58% of the cases over the disease course.^1^, ^11^ There is more ocular than orbital involvement, and the most common ocular manifestations are episcleritis and scleritis.^11^ The most common type of scleritis observed in patients with GPA is diffuse anterior and necrotizing scleritis, more rarely posterior scleritis.^12^ Association between ocular and neurological involvement is infrequent.^8^, ^11^ Contrary to our case, Bischof and al observed that in a large cohort study of 955 patients with AAV, the vasculitic neuropathy was associated with skin and musculo‐skeletal involvement instead of renal and ocular involvement.^8^

In case of suspicion of vasculitic neuropathy, extraneural biopsies (renal, nasal) can give inconclusive results, as observed here. Nasal biopsies yield low results, ranging from 16% to 53%.^13^ Nasal biopsies should be guided by nasal endoscopic findings such as bloody submucosal ulcers or persistent white submucosal nodules. These findings have a better reliability when related to histopathologic lesions of active GPA.^13^ Biopsies of the affected nerves may be the key to confirm the diagnosis, even more so when there is no clear evidence of extraneural vasculitis. The patient's nerve biopsy showed perivasculitis, which is compatible with vasculitic neuropathy but not typical. This can be linked to the nerve biopsy sensitivity. Indeed, nerve biopsy yields a sensitivity of about 50%^8^, ^14^ and reaches only 55% when associated with muscle biopsy.^14^ Sensitivity remains low due to these lesions being mostly focal.^8^ Hence, in clinical practice, nerve biopsy is not systematically performed.^8^ Typical pathological findings found in nerve biopsies are vessels obstruction with fibrinoid necrosis, inflammation of the vascular structures with structural damage, and granulomas. Axonal degeneration is also an additional suggestive finding.^14^ Despite the nonspecific histopathological findings, this case met the Chapel Hill nomenclature criteria for GPA, and the 2022 ACR/EULAR classification criteria. This is a combined score based on clinical, biologic, and histopathologic findings. Five or more points are necessary for the GPA diagnosis, with 93% sensitivity and 94% specificity.^15^, ^16^ Here, this case presented a total score of 9 points. Three points were attribute for nasal congestion history, 5 points for anti‐proteinase‐3 ANCA positivity, and 1 point for inflammation of the nasal/paranasal sinuses on imaging.^15^

Another point of discussion regards COVID‐19 association with AAV. We screened the available patient's blood samples. ANCA positivity was found on a serum sample prior to COVID‐19 disease. In June 2020, anti‐PR3 levels were 120 U/ml. We can support the hypothesis that the SARS‐CoV‐2 infection acted as a trigger for the GPA development, in a predisposing condition. There are two main processes by which infectious agents may induce auto‐immune disease: promoting immune system hyperstimulation and by molecular mimicry mechanisms.^17^ SARS‐CoV‐2 stimulates the immune system by inducing auto‐antibodies synthesis and triggers pre‐existing auto‐immune disease.^17^ For example, Vlachoyiannopoulos et al.^18^ described the presence of cANCA in 6,9% of patients in a 29‐patient cohort with severe COVID‐19 disease, and no history of auto‐immune disorder. Follow‐up studies are required in order to determine whether there is a clinical correlation with these antibodies, or if they are only transitory. In this case, pre‐existing ANCA antibodies prior to SARS‐CoV‐2 infection argue against a true seroconversion. Rather, it suggests pre‐existing disease activation. A few cases of new onset AAV after COVID‐19 disease have been reported, including notably three cases with GPA and lung involvement, and two of them also had other organs damage (e.g., kidneys, skin).^19^, ^20^, ^21^

Treatment strategy for SNV consists of induction and maintenance therapy. Induction treatment depends on the Five‐Factor Score (FFS). This tool assesses the prognosis of systemic necrotizing vasculitis (SNV) at initial diagnosis.^22^ For GPA, this score takes into consideration several organ involvements like renal, gastrointestinal, cardiac, and ear, nose, and throat (ENT) involvement as well as patient age. Each item is given +1 point. As ENT symptoms are associated with a better outcome, and consequently their absence is consequently scored +1 point. Presence of one or more factors is associated with a higher risk of death.^22^ Among these patients, a treatment with a combination of glucocorticoids and intravenous cyclophosphamide or rituximab is indicated.^23^ However, in GPA, contrasting with other SNV, it is recommended to immediately start a combination of steroids and immunosuppressant agents for systemic forms, regardless of the FFS, as there is a significant risk of relapse.^1^, ^2^, ^22^

We also consider peripheral neuropathy although it is not life‐threatening and not associated with higher mortality rates.^6^ Indeed, peripheral neuropathy can lead to disability and reduced quality of life.^6^ The results from the European Vasculitis Study Group trial show that all patients (MPA and GPA) with active vasculitic neuropathy at baseline achieved remission from their neuropathy with aggressive immunosuppressant therapy (mostly cyclophosphamide). Unfortunately, 65% demonstrated sequelae.^6^ Nerve recovery is known to be slow. For all these reasons, and according to these guidelines, we have treated this patient with a reduced‐dose regimen of steroids and rituximab. A reduced‐dose regimen of glucocorticoids was motivated in order to avoid frequently reported side effects of steroids, particularly corticosteroid‐induced myopathy in a context of neuropathy. In this regard, a recent Japanese randomized non‐inferiority trial compared two steroids regimens (reduced vs conventional high dose) in addition to rituximab in 140 patients with AAV. There was no evidence of a difference in remission rates at 6 months, while a lower rate of serious infections was noticed for the reduced regimen.^24^ Similarly, the PEXIVAS trial is a recent study which included patients with AAV with renal involvement or severe diffuse hemorrhage.^25^ Patients were randomized to either receive or not plasma exchange, and a standard or reduced‐dose regimen of steroids. Walsh and al. did not demonstrate any effectiveness of the plasma exchange in terms of mortality or incidence of end‐stage kidney disease. However, they demonstrated non‐inferiority of the reduced‐dose regimen of glucocorticoids, and a lower incidence of serious infections at 1 year.^25^

It is increasingly accepted that total steroids dose during remission induction phase could be reduced in most patients with AAV, regardless of concomitant immunosuppressive therapy. GPA is known to have a high relapse risk,^1^ especially with anti‐PR3 ANCA positivity.^2^ Hence, maintenance therapy is recommended with Azathioprine or Rituximab. Of note, the MAINRITSAN trial showed rituximab superiority over azathioprine, with reduced disease relapse,^26^ supporting our choice of Rituximab‐based maintenance therapy.

## 4 CONCLUSION

ANCA‐vasculitis is a systemic disorder characterized by granulomatous inflammation of different tissues, and small vessels necrotizing vasculitis, and has several clinical presentations.^1^ This case demonstrates the importance of peripheral neuropathy identification, as an initial clinical presentation of GPA. This allows early diagnosis and prompt immunosuppressive treatment initiation, preventing (or at least, delaying) any life‐threatening organ involvement. Diagnosis is usually evident, based on clinical aspects and serological ANCA testing. However, biopsies may be needed, and remain the gold standard.^1^, ^27^ In this case, nasal and nerve biopsies showed perivasculitis without granulomas. Due to these focal lesions, granulomas are frequently missing in the biopsies.^8^, ^13^

Viral infections can trigger GPA.^1^ Here, SARS‐CoV‐2 infection may have triggered disease development. To our knowledge, there are few published cases of new onset granulomatosis with polyangiitis associated with COVID‐19.^19^, ^20^, ^21^ Most of these findings were published as case reports, cohort, or prospective studies are needed to identify rheumatologic manifestations in the short‐ and long‐term following COVID‐19 infection.

## AUTHOR CONTRIBUTIONS

Dr Weynand Marjolaine: Substantial contributions to the drafting of the article, acquisition and analysis of the data. Dr Raftakis Ioannis: Critical revising of the article. Dr Chérif Mohammad Yassine: Critical revising of the article. Dr Lecomte Sophie: acquisition of the data and analysis. Pr Badot Valerie: Critical revising of the article and final approval of the version to be published.

## CONFLICT OF INTEREST

There is no conflict of interest for any authors.

## CONSENT

Written informed consent was obtained from the patient to publish this report in accordance with the journal's patient consent policy.

## Data Availability

Data sharing is not applicable to this article as no new data were created or analyzed in this study.

## References

[1] Comarmond C , Cacoub P . Granulomatosis with polyangiitis (Wegener): clinical aspects and treatment. Autoimmun Rev. 2014;13(11):1121‐1125. 25149391 10.1016/j.autrev.2014.08.017

[2] Puéchal X , Pagnoux C , Perrodeau É , et al. Long‐term outcomes among participants in the WEGENT trial of remission‐maintenance therapy for granulomatosis with polyangiitis (Wegener's) or microscopic polyangiitis. Arthritis Rheumatol. 2016;68(3):690‐701. 26473755 10.1002/art.39450

[3] Wolf J , Schmitt V , Palm F , Grau AJ , Bergner R . Peripheral neuropathy as initial manifestation of primary systemic vasculitides. J Neurol. 2013;260(4):1061‐1070. 23212754 10.1007/s00415-012-6760-7

[4] Nishino H , Rubino FA , DeRemee RA , Swanson JW , Parisi JE . Neurological involvement in Wegener's granulomatosis: an analysis of 324 consecutive patients at the Mayo clinic. Ann Neurol. 1993;33(1):4‐9. 8388187 10.1002/ana.410330103

[5] de Groot K , Schmidt DK , Arlt AC , Gross WL , Reinhold‐Keller E . Standardizaed neurologic evaluations of 128 patients with Wegener granulomatosis. Arch Neurol. 2001;58:1215‐1221. 11493161 10.1001/archneur.58.8.1215

[6] Suppiah R , Hadden RD , Batra R , et al. Peripheral neuropathy in ANCA associated vasculitis: outcomes from the European Vasculitis study group trials. Rheumatology. 2011;50:2214‐2222. 21890618 10.1093/rheumatology/ker266

[7] Imboden JB . Involvement of the peripheral nervous system in polyarteritis nodosa and antineutrophil cytoplasmic antibodies–associated Vasculitis. Rheum Dis Clin N Am. 2017;43(4):633‐639. 10.1016/j.rdc.2017.06.011 29061248

[8] Bischof A , Jaeger VK , Hadden RD , et al. Peripheral neuropathy in antineutrophil cytoplasmic antibody‐associated vasculitides: insights from the DCVAS study. Neurol Neuroimmunol Neuroinflamm. 2019;6(6):e615. 31540965 10.1212/NXI.0000000000000615 PMC6807658

[9] Mills KR . The basics of electromyography. J Neurol Neurosurg Psychiatry. 2005;76(suppl 2):ii32‐ii35. 15961866 10.1136/jnnp.2005.069211 PMC1765694

[10] Csernok E , Moosig F . Current and emerging techniques for ANCA detection in vasculitis. Nat Rev Rheumatol. 2014;10:494‐501. 24890776 10.1038/nrrheum.2014.78

[11] Asin MA , Charles P , Rothschild P‐R , Terrier B . Ocular involvement in granulomatosis with polyangiitis: a single‐center cohort study on 63 patients. Autoimmun Rev. 2019;18(2019):493‐500. 30844550 10.1016/j.autrev.2019.03.001

[12] Cocho L , Gonzalez‐Gonzalez LA , Molina‐Prat N , Doctor P , Sainz‐de‐la‐Maza M , Foster CS . Scleritis in patients with granulomatosis with polyangiitis (Wegener). Br J Ophtalmol. 2016;100:1062‐1065. 10.1136/bjophthalmol-2015-307460 26567022

[13] Boltrán Rodriguez‐Cabo O , Reyes E , Rojas‐Serrano J , Flores‐Suárez LF . Increased histopathological yield for granulomatosis with polyangiitis based on nasal endoscopy of suspected active lesions. Eur Arch Otorhinolaryngol. 2018;275(2):425‐429. 29230559 10.1007/s00405-017-4841-z

[14] Nathani D , Spies J , Barnett MH , et al. Nerve biopsy: current indications and decision tools. Muscle Nerve. 2021;64(2):125‐139. 33629393 10.1002/mus.27201 PMC8359441

[15] Robson JC , Grayson PC , Ponte C , et al. 2022 American College of Rheumatology/European Alliance of associations for rheumatology classification criteria for granulomatosis with polyangiitis. Arthritis Rheumatol. 2022;74(3):386‐392. 35106968 10.1002/art.41982

[16] Jennette JC , Falk RJ , Bacon PA , et al. 2012 revised international Chapel Hill consensus conference nomenclature of Vasculitides. Arthritis Rheum. 2013;65:1‐11. 23045170 10.1002/art.37715

[17] Dotan A , Muller S , Kanduc D , David P , Halpert G , Shoenfeld Y . The SARS‐CoV‐2 as an instrumental trigger of autoimmunity. Autoimmun Rev. 2021;20:102792. 33610751 10.1016/j.autrev.2021.102792 PMC7892316

[18] Vlachoyiannopoulos PG , Magira E , Alexopoulos H , et al. Autoantibodies related to systemic autoimmune theumatic diseases in severely ill patients with COVID‐19. Ann Rheum Dis. 2020;79:1661‐1663. 32581086 10.1136/annrheumdis-2020-218009

[19] Hussein A , Khalil KA , Bawazir YM . Anti‐neutrophilic cytoplasmic antibody (ANCA) vasculitis presented as pulmonary hemorrhage in a positive COVID‐19 patient: a case report. Cureus. 2020;12(8):e9643. 32923243 10.7759/cureus.9643 PMC7480889

[20] Selvaraj V , Moustafa A , Dapaah‐Afriyie K , Birkenbach MP . COVID‐19‐induced granulomatosis with polyangiitis. BMJ Case Rep. 2021;14(e242142):5. 10.1136/bcr-2021-242142 PMC7978249 33737283

[21] Bressler MY , Pathak N , Cervellione K , et al. New onset granulomatosis with polyangiitis associated with COVID‐19. Case Rep Dermatol Med. 2021;2021:8877292. 33505734 10.1155/2021/8877292 PMC7811565

[22] Guillevin L , Pagnoux C , Seror R , et al. The five‐ factor score revisited: assessment of prognoses of systemic necrotizing vasculitides based on the French Vasculitis study group cohort. Medicine. 2011;90:19‐27. 21200183 10.1097/MD.0b013e318205a4c6

[23] Stone JH , Merkel PA , Spiera R , et al. Rituximab versus cyclophosphamide for ANCA‐associated vasculitis. N Engl J Med. 2010;363(3):221‐232. 20647199 10.1056/NEJMoa0909905 PMC3137658

[24] Furuta S , Nakagomi D , Kobayashi Y , et al. Effect of reduced‐dose vs high‐dose glucocorticoids added to rituximab on remission induction in ANCA‐associated vasculitis: A randomized clinical trial. JAMA. 2021;325:2178‐2187. 34061144 10.1001/jama.2021.6615 PMC8170547

[25] Walsh M , Merkel PA , Peh CA , et al. Plasma exchange and glucocorticoids in severe ANCA‐associated Vasculitis. N Engl J Med. 2020;382:622‐631. 32053298 10.1056/NEJMoa1803537 PMC7325726

[26] Guillevin L , Pagnoux C , Karras A , et al. Rituximab versus azathioprine for maintenance in ANCA‐associated vasculitis. N Engl J Med. 2014;371(19):1771‐1780. 25372085 10.1056/NEJMoa1404231

[27] Yates M , Watts RA , Bajema IM , et al. EULAR/ERA‐EDTA recommendations for the management of ANCA‐associated vasculitis. Ann Rheum Dis. 2016;75(9):1583‐1594. 27338776 10.1136/annrheumdis-2016-209133

